# Supplementary material for: Complex‐centric proteome profiling by SEC‐SWATH‐MS
Source: Mol Syst Biol. 2019 Jan 14;15(1):e8438. doi: 10.15252/msb.20188438 (PMC6346213; doi:10.15252/msb.20188438)
Supplement: Supplementary file 6 — Dataset EV5 [file MSB-15-e8438-s006.zip › feature_plots_corum/486.pdf]

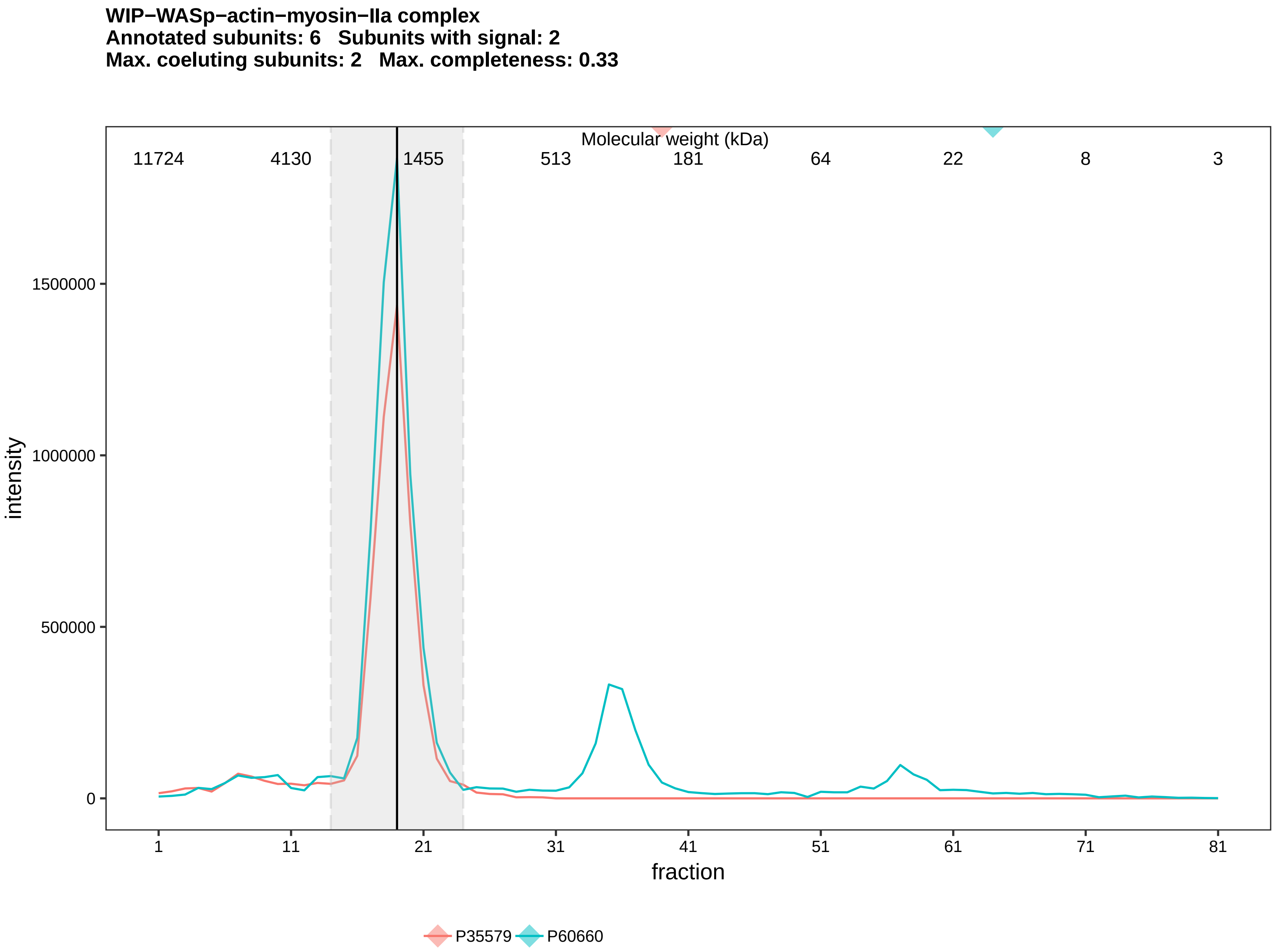
WIP−WASp−actin−myosin−IIa complex
Annotated subunits: 6 Subunits with signal: 2
Max. coeluting subunits: 2 Max. completeness: 0.33
Molecular weight (kDa)
11724
4130
1455
513
181
64
22
8
3
1500000
1000000
500000
0
intensity
1
11
21
31
41
51
61
71
81
fraction
P35579
P60660
